# Daring to be differential: metabarcoding analysis of soil and plant-related microbial communities using amplicon sequence variants and operational taxonomical units

**DOI:** 10.1186/s12864-020-07126-4

**Published:** 2020-10-22

**Authors:** Lisa Joos, Stien Beirinckx, Annelies Haegeman, Jane Debode, Bart Vandecasteele, Steve Baeyen, Sofie Goormachtig, Lieven Clement, Caroline De Tender

**Affiliations:** 1 Flanders Research Institute for Agriculture, Fisheries and Food (ILVO), Plant Sciences Unit, Burgemeester Van Gansberghelaan 92, 9820 Merelbeke, Belgium; 2 Department of Applied Mathematics, Computer Science and Statistics, Ghent University, Krijgslaan 281, 9000 Ghent, Belgium; 3 Department of Plant Biotechnology and Bioinformatics, Ghent University, Technologiepark 71, 9052 Ghent, Belgium; 4 Center for Plant Systems Biology, VIB, Ghent, Technologiepark 71, 9052 Ghent, Belgium

**Keywords:** Soil, Rhizosphere and endosphere microbiome, Metabarcoding analysis, OTU, ASV

## Abstract

**Background:**

Microorganisms are not only indispensable to ecosystem functioning, they are also keystones for emerging technologies. In the last 15 years, the number of studies on environmental microbial communities has increased exponentially due to advances in sequencing technologies, but the large amount of data generated remains difficult to analyze and interpret. Recently, metabarcoding analysis has shifted from clustering reads using Operational Taxonomical Units (OTUs) to Amplicon Sequence Variants (ASVs). Differences between these methods can seriously affect the biological interpretation of metabarcoding data, especially in ecosystems with high microbial diversity, as the methods are benchmarked based on low diversity datasets.

**Results:**

In this work we have thoroughly examined the differences in community diversity, structure, and complexity between the OTU and ASV methods. We have examined culture-based mock and simulated datasets as well as soil- and plant-associated bacterial and fungal environmental communities. Four key findings were revealed. First, analysis of microbial datasets at family level guaranteed both consistency and adequate coverage when using either method. Second, the performance of both methods used are related to community diversity and sample sequencing depth. Third, differences in the method used affected sample diversity and number of detected differentially abundant families upon treatment; this may lead researchers to draw different biological conclusions. Fourth, the observed differences can mostly be attributed to low abundant (relative abundance < 0.1%) families, thus extra care is recommended when studying rare species using metabarcoding. The ASV method used outperformed the adopted OTU method concerning community diversity, especially for fungus-related sequences, but only when the sequencing depth was sufficient to capture the community complexity.

**Conclusions:**

Investigation of metabarcoding data should be done with care. Correct biological interpretation depends on several factors, including in-depth sequencing of the samples, choice of the most appropriate filtering strategy for the specific research goal, and use of family level for data clustering.

## Background

Microorganisms play a number of key roles in soil ecosystem functions [1]. Unraveling the microbial communities that reside in soil systems and on plant root-soil interfaces is important for increasing sustainability in agriculture [2–4]. Tremendous progress in DNA sequencing technologies has allowed for analysis and insight into the composition and behavior of microbial communities in various environments. Currently, metabarcoding is often used to gain insight into the structure and dynamics of microbial communities through analysis of the relative abundances and taxonomical diversity within samples [5, 6]. With the advent of these technologies, the newest research challenges are in the area of data analysis and interpretation.

Recently, the shift from the use of Operational Taxonomical Units (OTUs) to Amplicon Sequence Variants (ASVs) has been one of the major modifications in classification of DNA sequences when analyzing metabarcoding experiments [7, 8]. In OTU analysis, microbial DNA sequences are clustered together in one OTU by means of a similarity threshold (usually 97%), assigning sequences to species and higher taxonomic levels [9, 10]. This approach has historically dominated the field. Various algorithms and thresholds can be used to calculate OTUs, with the use of diverse microbiome analysis tools such as QIIME, UPARSE and Mothur [11–13]. Although OTU calling consolidates sequences that differ because of sequencing and amplification errors, it cannot account for small biological variations. Taxonomically different sequences can be clustered into one OTU, but this can disregard the real biological sequence variation. Recent data analysis methods have attempted to overcome this drawback by determining the output reads into ASVs [14]. ASV workflows partition the reads based on error models to correct sequencing errors while also accounting for abundance and sequence similarity [7]. In this way, ASV calling can detect small biological sequence variants and discard technical errors introduced by library preparation and sequencing technology, this increases the taxonomical resolution of the results [15]. The possibility does exist, however, that real biological variations are classified as technical errors, especially when data quality is insufficient or the dataset is too small.

**Fig. 1:**
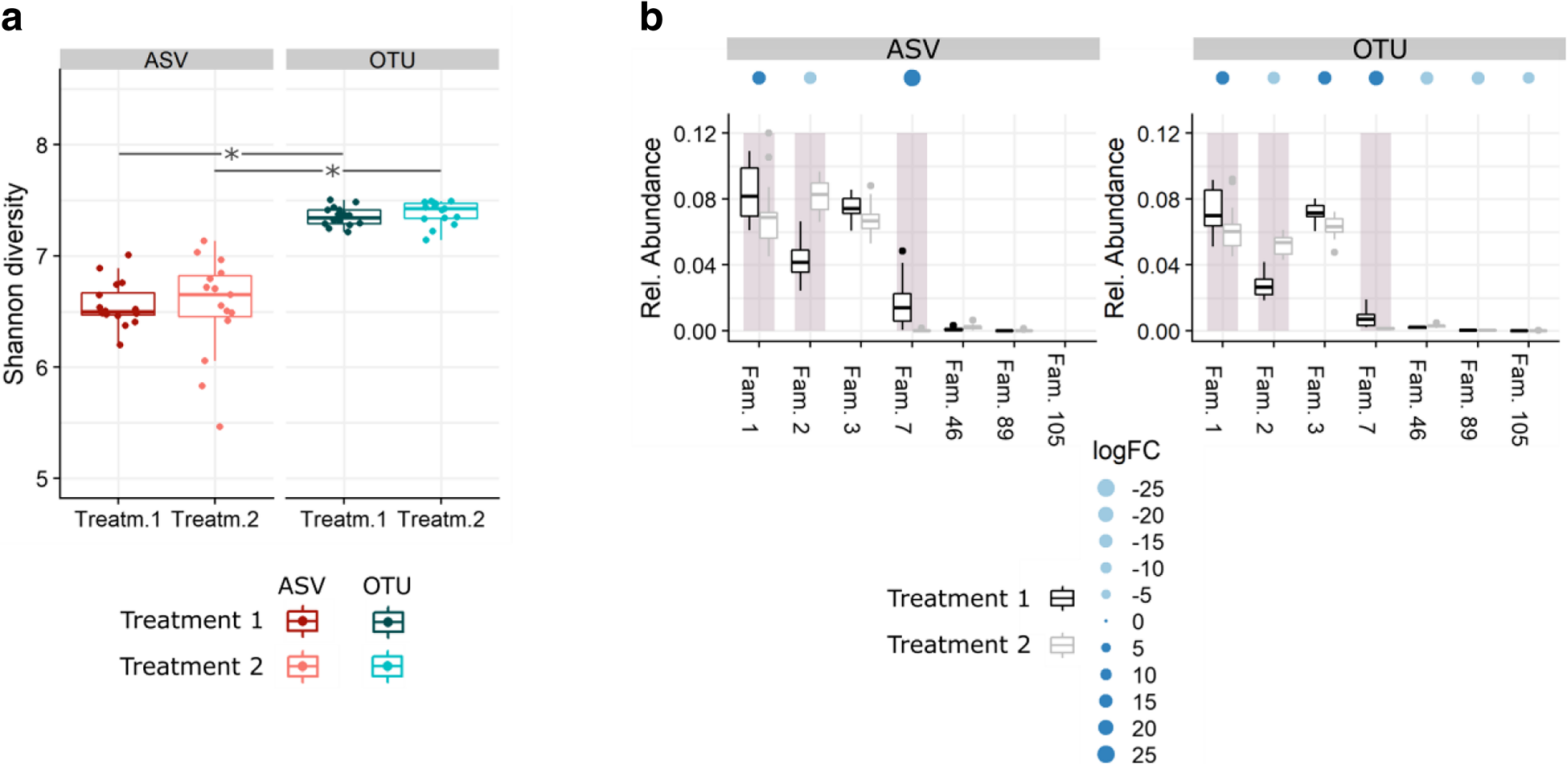
Differences between the ASV and OTU methods for Shannon diversity and differentially abundant families in a bacterial soil dataset. **a** Shannon diversity per treatment (treatments 1 and 2) for each method. Samples are displayed as dots (*n* = 16). Asterisks indicate significant differences between diversity measurements (*P* < 0.05). **b** Selected families of the bacterial soil dataset with their respective relative abundance per treatment for the ASV and OTU methods. The presence of dots above the families indicate a significant effect of the applied treatment (FDR 5%) and the dot size corresponds to the logFC. The light and dark blue color marks a decrease or an increase in relative abundance, respectively. The gray boxes are families found to be significant in both methods

The choice of either a clustering or error-model based method will affect the biological interpretation and the conclusions of metabarcoding experiments [15–19]. The choice of method also strongly affects the reported microbial diversity and richness. Previous research has confirmed that OTU and ASV methods are not always consistent for alpha diversity measurements [19, 20], but despite this clearly documented influence, variations in differential abundances caused by treatments or sample types have not yet been systematically analyzed (Fig. 1a, b).

Our aim was to dissect the assessment differences in community diversity, structure, and complexity and to describe their influence on the formulation of biological conclusions when using either an OTU vs. ASV method to analyze the datasets. We based our analysis on two of the most prominent methods in the field: an OTU-clustering method using the USEARCH-UPARSE workflow and an error-model based ASV method by the DADA2 workflow [14, 21]. We hypothesized that the chosen method affects alpha diversity measurements and detected relative abundances, leading to different biological interpretations of the same datasets. Additionally, we hypothesized that the different occurrences between methods could be due to either (i) specific settings in the preprocessing or (ii) the fundamental variations in the read partitioning, i.e., threshold (OTU) vs. error model (ASV).

To illustrate these differences, we first analyzed bacterial and fungal culture-based microbial communities and simulated datasets. These datasets were then used to validate the performance and taxonomic resolution of both methods. In addition, we investigated the differences in biological interpretation of bacterial and fungal soil- and plant-related microbial communities at family level as analyzed using both the OTU and ASV methods. These soil- and plant-related datasets have higher diversity and are less well identified than the human-related communities that are often used to benchmark metabarcoding workflows [11, 14]. For the soil and plant communities, the effect of non-inversion vs. conventional tillage and rhizosphere vs. endosphere were analyzed, respectively.

## Results

**Fig. 2:**
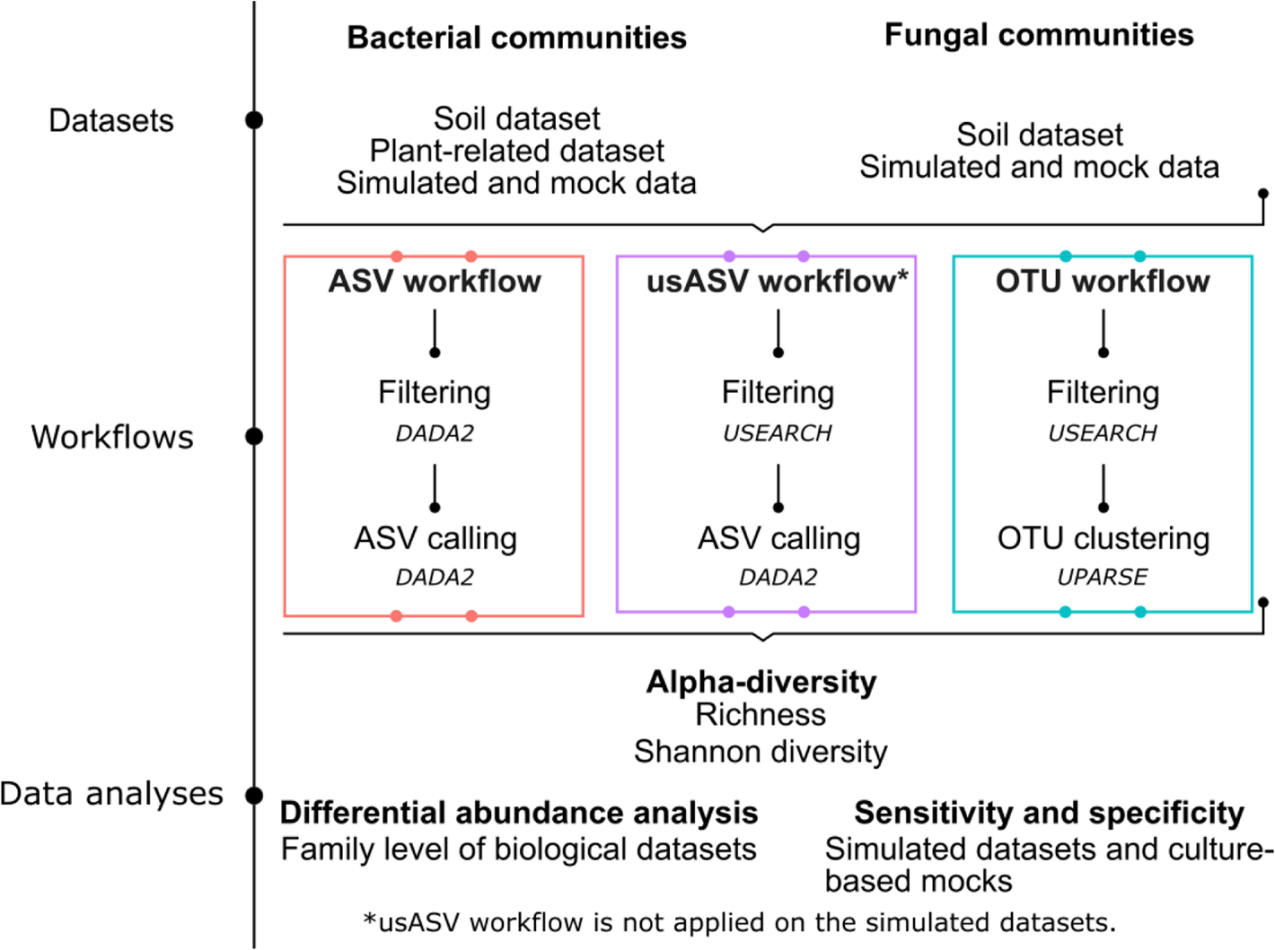
Overview of datasets, methods, and data analyses used

We compared an OTU calling (97% similarity threshold) and an ASV (error model) method by first validating the performance and sensitivity of the methods using simulated and culture-based mock datasets of bacterial and fungal communities. Next, we studied the alpha diversity and differential abundances of bacterial and fungal communities in soil and plant-related datasets. To distinguish between differences caused by preprocessing or read partitioning, an additional workflow (usASV) was developed that combines the USEARCH filtering and merging with the ASV error model (Fig. 2) that was used for the culture-based mock and biological datasets. No sequencing or PCR errors were introduced into the simulated mock dataset, showing that the results of the usASV were similar to those of the ASV and warranted no further discussion.

### ASV and OTU methods in simulated dataset and culture-based mock bacterial communities

The sensitivity of both methods was first validated using a simulated dataset of the bacterial community. In total, 20 bacterial communities were simulated from the SILVA 16S rRNA gene reference database [22]. Simulated libraries varied from low (100 or 500 unique sequences) to high community richness (1000 or 2500 unique sequences) with alternating sequencing depth. These simulated datasets were analyzed using the OTU, ASV, and usASV methods.

**Fig. 3:**
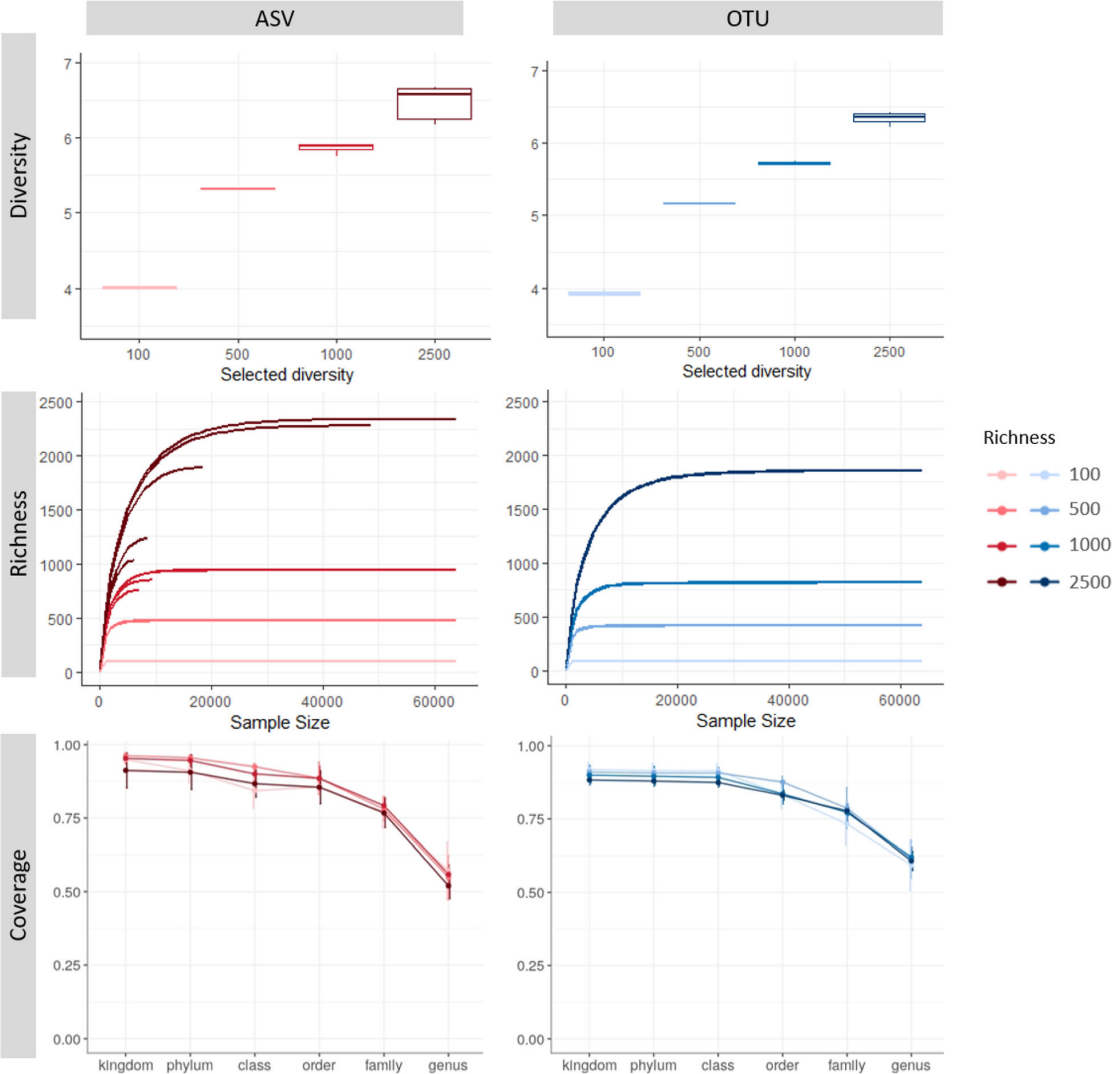
Representation of the species richness, diversity, and coverage for the simulated bacterial dataset either analyzed by the ASV method (red) or OTU method (blue). Datasets are simulated from the SILVA 16S rRNA gene database with an original community richness varying between 100 (light colored) and 2500 (dark colored). Top panels, Shannon diversity index per original sample richness; middle panels, community richness with increasing sequencing depth; the bottom panels, coverage of each method per taxonomic level

Community richness (defined by the number of ASVs) and diversity (measured by the Shannon diversity index) were both slightly lower in the OTU method than in the ASV method for all sequencing depths in the simulated datasets (Fig. 3). In both methods, richness was estimated quite accurately in samples with a low community richness. For a true 100 species richness, the OTU and ASV methods estimated a richness of 89.0 ± 1 and 95.0 ± 1, respectively, whereas for a true 500 species richness, 475.0 ± 3 ASVs and 414.1 ± 2 OTUs were found. In samples with higher true richness, both methods underestimated the richness (Fig. 3). For a true richness of 1000 species, the OTU and ASV methods estimated a richness of 800 ± 19 and 885 ± 82, respectively. For a richness of 2500 sequences, 1678 ± 205 OTUs and 1753 ± 595 ASVs were estimated. However, when sequencing depth was high enough (> 50,000 sequences), the ASV method outperformed the OTU method in the samples with higher community richness. Similarly, sequencing depth and sample richness were highly correlated in the ASV method, leading to an underestimation of the community richness at lower sequencing depths in diverse bacterial communities (Fig. 3). Additionally, a plateau in the richness curves was noticed for both the ASV and OTU methods.

Besides alpha diversity, we also studied the community composition of the simulated datasets (Fig. 3). For data aggregated at family level or higher, both methods performed equally well in assigning the correct taxonomy, with over 75% of the OTUs/ASVs being correctly identified. At genus level, the coverage decreased in both methods to below 50% for the ASV method. This might be due to a higher number of unclassified ASVs (39.7% ± 6.1%) in the low-level taxonomy than the number of unclassified OTUs (9.1% ± 2.8%) (Fig. 3).

A bacterial culture-based mock dataset was used to better resemble environmental samples. These culture-based mock communities were obtained by pooling 254 strains from a bacterial collection before DNA extraction and sequencing. In addition to the OTU and ASV method, the usASV method was applied to the culture-based mock. Similar to the observations of simulated data, the richness and diversity of OTUs were remarkably lower than those of ASVs (Fig. S1 A, B). This is likely due to sequences of different strains that are clustered together as one OTU in the culture-based mock. In total, 84 ± 4 ASVs, 45 ± 1 OTUs, and 82 ± 4 usASVs were identified instead of the actual 254 strains (Fig. S1B). In addition, the taxonomy assignment of the culture-based mock communities (V3-V4 region) was compared with the taxonomy determined on the complete 16S rRNA gene of the isolated strains in the bacterial collection. The number of correctly identified bacterial taxa, false positives, and false negatives were similar to family level, i.e., 15 of the 22 families were identified in all three methods. Only at genus level, the OTU method performed slightly better than the ASV method (22 of the 31 genera were identified, one more than for the ASVs) (Table S1).

In conclusion, the ASV method made a better estimation of sample richness and diversity for both low and high diversity samples compared to the OTU method when the sequencing depth is high. The performance of both methods is comparable for taxonomical classification up to family level.

### Differences between ASV and OTU methods in soil-related bacterial communities

The bacterial community was isolated from samples of Belgian field soil where non-inversion and conventional tillage were applied [23]. These communities are referred to below as the soil dataset. This dataset was studied by applying either ASV, usASV, or OTU methods. The unfiltered ASV method supplied fewer ASVs than OTUs, although the difference was rather limited (11,492 ASVs vs. 13,407 OTUs); in contrast, the usASV method was characterized by 21,282 usASVs (Table S2). After application of technical filtering to remove spurious sequences (fewer than two counts in at least three independent samples), a decrease of approximately 70% in the number of ASVs detected for both the ASV and usASV methods. This was mainly due to the removal of unique sample sequences, in contrast to the number of OTUs (decrease of 15%) (Table S2).

**Fig. 4:**
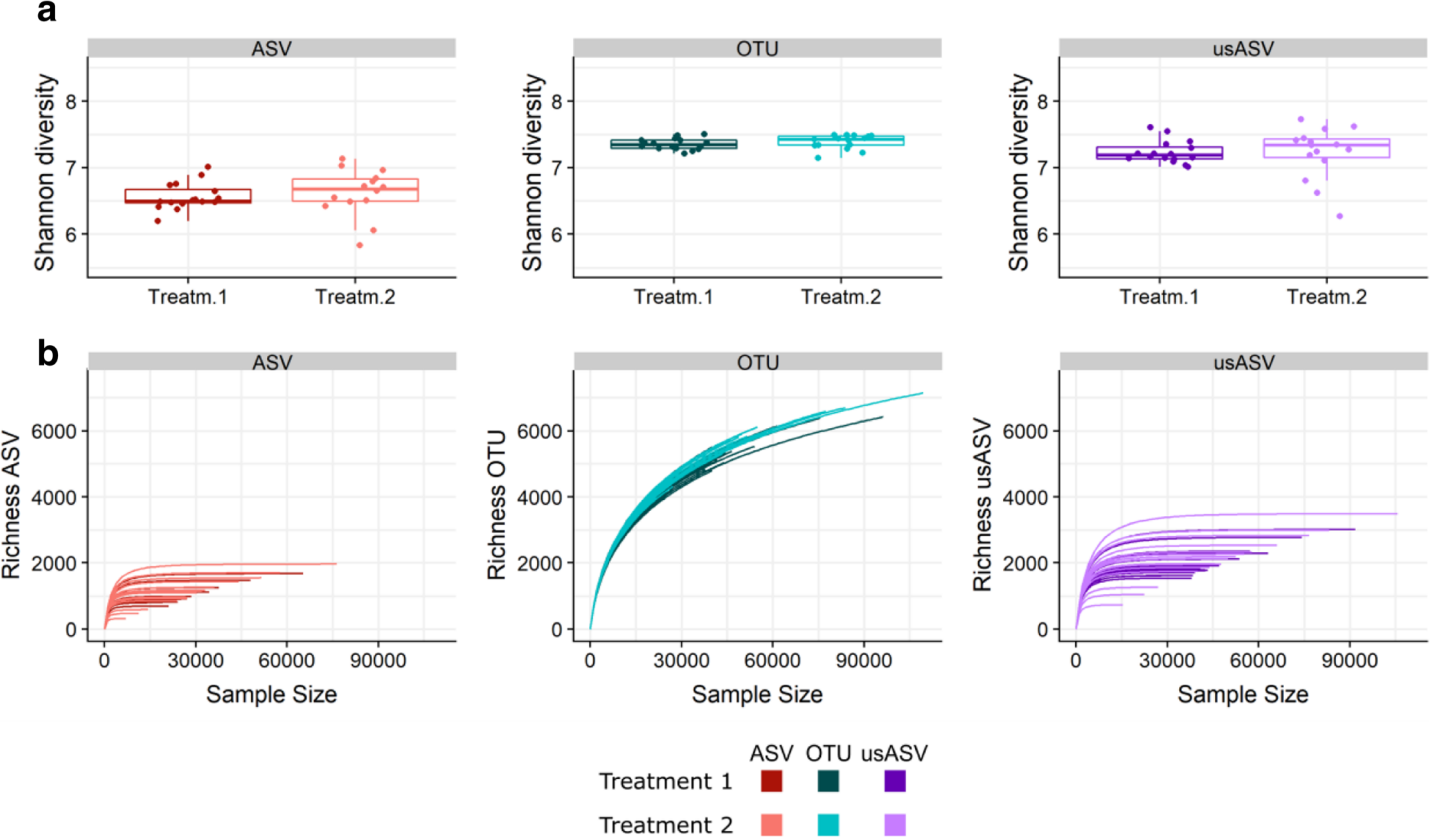
Shannon diversity and richness versus sequencing depth of ASV, OTU, and usASV methods in the bacterial soil dataset. **a** Shannon diversity for Treatment 1 (non-inversion tillage) versus Treatment 2 (conventional tillage) for each method. Samples are displayed as dots (*n* = 16). **b** For each method, richness with increasing sequencing depth (*n* = 16) for both treatments

In contrast to our observations of the simulated dataset and culture-based mock, the community diversity of the soil dataset was significantly lower using the ASV method (6.55 ± 0.34) than the OTU (7.37 ± 0.1) and usASV (7.23 ± 0.29) method (*P* < 0.001) (Figs. 1 and 4a). In addition, the community richness of ASVs was on average five-fold (*P* < 0.001) and two-fold (*P* < 0.001) lower than that of the OTUs and usASVs, respectively (Fig. 4b). The usASVs were still two-fold lower than of the OTU output (*P* < 0.001), indicating less strict merging and filtering of the OTU method. Remarkably, the ASV richness reached a plateau after a sequencing depth of 12,000 sequences, whereas the OTU richness continued to increase, in contrast to the simulated data and culture-based mock, even beyond a sequencing depth of 125,000 sequences (Fig. 4b). Furthermore, the number of detected ASVs was correlated with the sequencing depth, i.e., the number of ASVs increased with enhanced sequencing depth as observed in the simulated data. The same correlation and curve flattening were observed for the usASVs but not for the OTUs; this hints at the influence of the ASV read partitioning (Fig. 4b). For all methods, neither Shannon diversity nor richness differed between treatments (*P* > 0.05).

An additional, more stringent abundance filtering was applied (at least two per sample in at least three independent samples) before the data were grouped at family level, in order to retain the best performing taxonomic resolution as identified with the simulated data and culture-based mock (Table S2). The resulting bacterial community composition was examined. Between the ASV and OTU method, 143 families overlapped, but 40 families were found exclusively in the OTU method. Two of the unique OTU families were also detected using the usASV method. The unique OTU families were all low abundant, ranging from 0.006 to 0.01% mean relative abundance (RA) in the bacterial community. Originally, most of these families occurred in the ASV dataset but had been removed by abundance filtering. Four families (*Algiphilaceae*, mle1–27, *Demequinaceae*, and *Isosphaeraceae*) were truly absent from the ASV dataset.

**Fig. 5:**
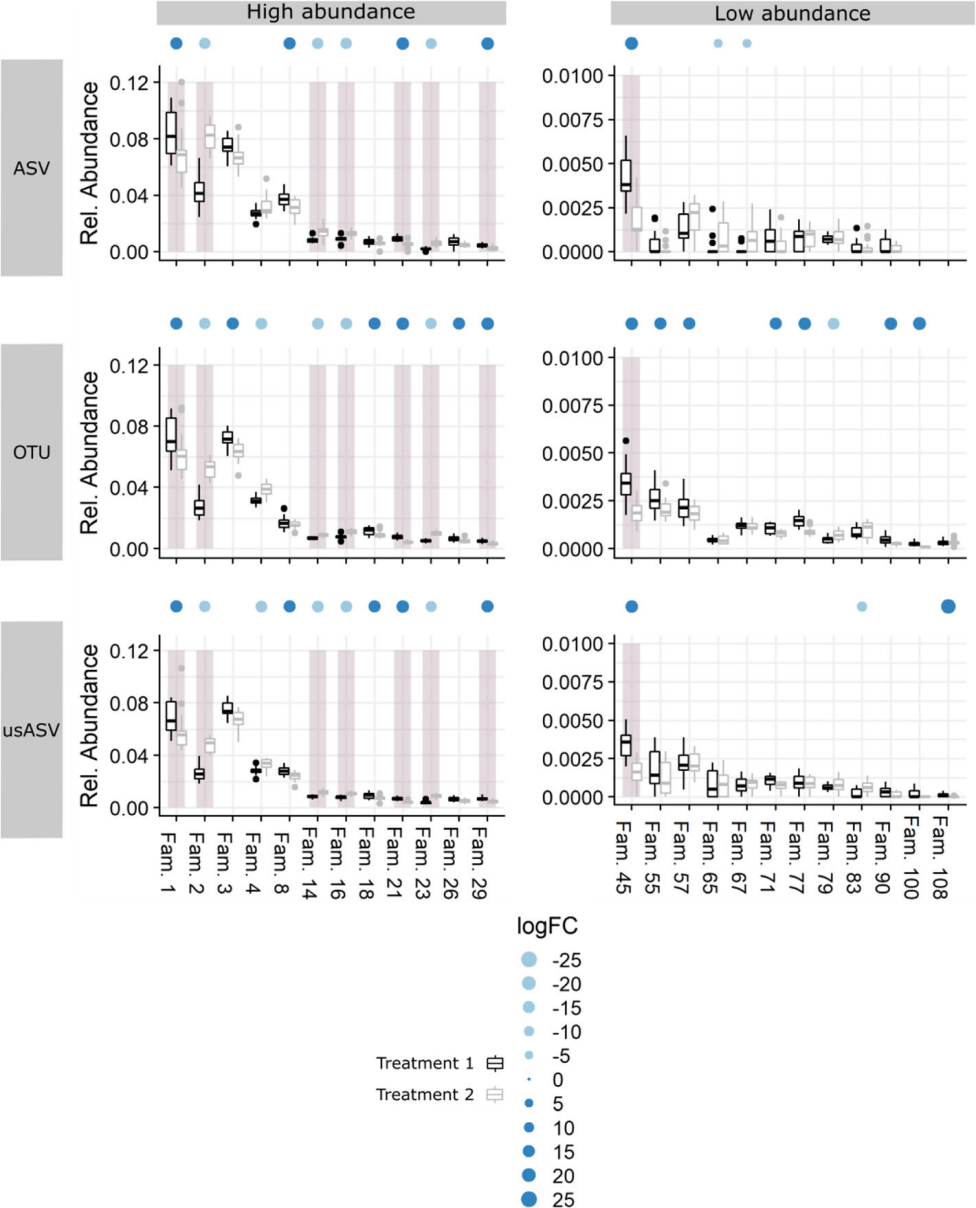
Differences between the ASV, OTU, and usASV methods in bacterial communities in the soil dataset. Selected families of both methods are shown with their respective relative abundance per treatment (*n* = 16) (for all significant families, see Fig. S2). The data are split into high (RA > 0.001) and low (RA < 0.001) relative abundance families. The dots above the families indicate a significant effect of the applied treatment (FDR 5%) and the dot size corresponds to the logFC. The light and dark blue colors mark decrease and increase in relative abundance, respectively. The gray boxes are families found significant in all three methods

Differential abundances at the family level, a result of different tillage practices, were compared for all methods (Fig. 5 and S2). For the ASV method, a significant difference between non-inversion tillage and conventional tillage was found for 30 families, 15 of which showed an increased RA when conventional tillage was applied (False Discovery Rate [FDR] < 0.05). Using the OTU method, conventional tillage significantly altered the RA of 104 families (FDR < 0.05) with increased RA for 58 families. Using the usASV method, RA of 58 families changed significantly with increased RA for 30 families (FDR < 0.05). The larger proportion of significant families in the OTU method was mainly due to low abundant families (RA < 0.1%). Between the ASV and OTU dataset, only 27 significant families overlapped; 23 of these families had an RA higher than 0.1% and displayed the same increase or decrease in RA (Fig. 5 and S2). Across all three methods, 23 families were significantly differentially abundant; these families were predominantly more abundant (22 families with RA > 0.1%) (Fig. S2). In comparison, 100 families were found to be high abundant (RA > 0.1%) across all methods. Of the 77 families with sole significance in the OTU method, 24 overlapped in the usASV method, illustrating that preprocessing (filtering and merging of USEARCH) was responsible for approximately 30% of the uniquely significant families present in the OTU method. Of all significant families in the OTU dataset, three families were completely absent in the ASV dataset.

As low abundant families caused the largest difference between both methods, the analysis was redone only on families with an RA higher than 0.1 and 0.5% (Table S3). When the 0.1% filtering was applied, 30 and 61 families were significantly differentially abundant for the ASV and OTU methods, respectively, with an overlap of 26 families. For the 0.5% filtering, 15 families overlapped, with 18 and 30 significantly differentially abundant families for the ASV and OTU methods, respectively.

Differences in the biological inference of the treatment effect were observed between the ASV and OTU methods. The reasons for these variations could, in part, be due to the filtering and merging of USEARCH, but could also be substantially the fundamental differences in the error model used for ASVs and the 97% similarity threshold for OTUs.

### Differences between ASV and OTU methods in plant-related bacterial communities

To confirm the results of the soil dataset and to evaluate the differences in metabarcoding methods on samples with reduced diversity, we examined the bacterial communities of a root-related dataset, referred to below as the plant dataset. This dataset contained bacterial sequences of two plant-related compartments, the rhizosphere, i.e., the soil closely surrounding the root, and the endosphere, i.e., the inside of the plant root of field soil-grown maize (*Zea mays* L.) [24].

Similar to the soil dataset, the detected number of ASVs was lower than OTUs, both before and after filtering (less than two counts in three independent samples) and after removal of plant-related reads (Table S2). In contrast, the number of ASVs detected using the usASV method was located between the number of the two other methods, both before and after the filtering steps (Table S2).

**Fig. 6:**
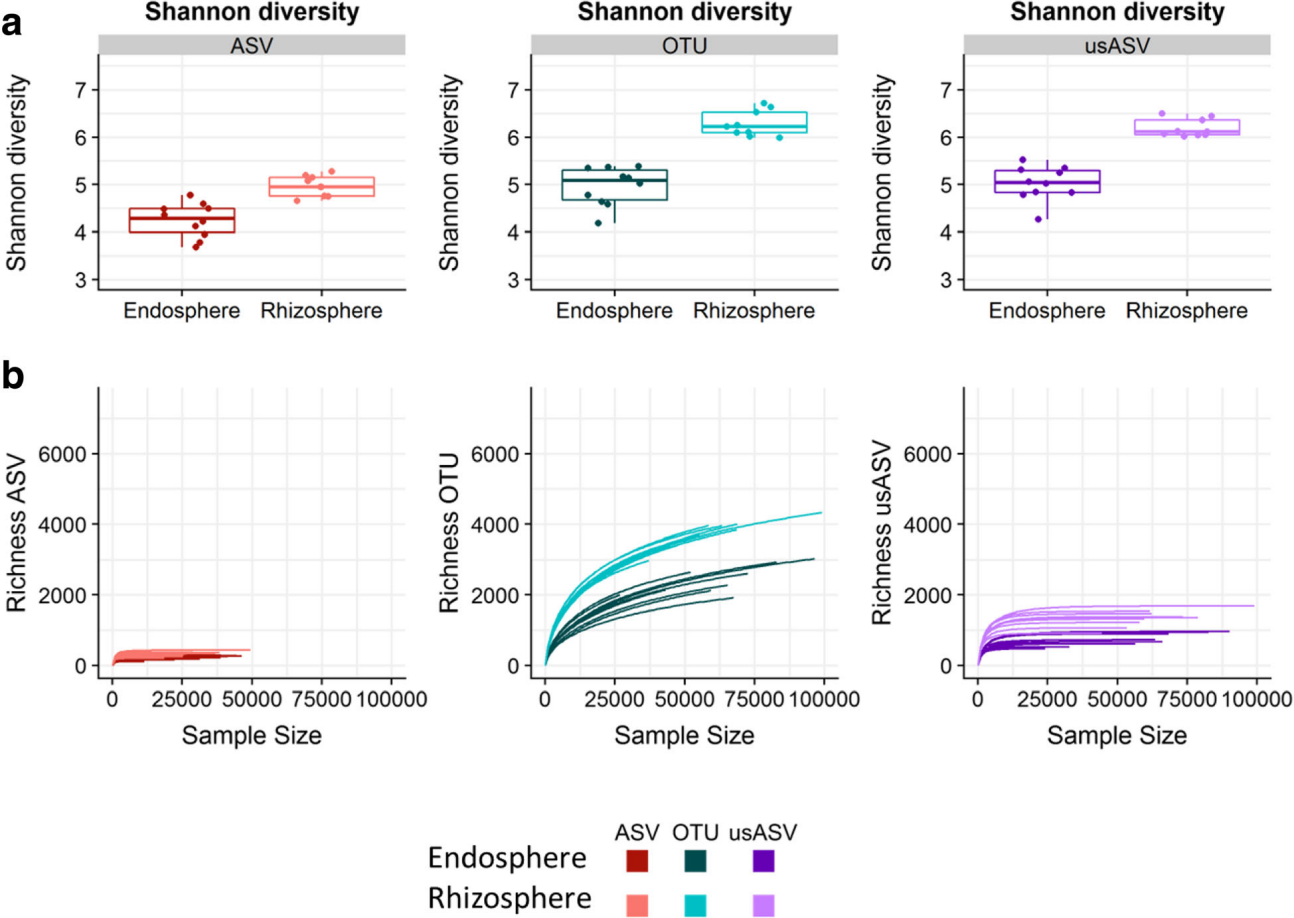
Shannon diversity and richness verus sequencing depth of ASV, OTU, and usASV methods in the bacterial plant dataset. **a** Shannon diversity per compartment, rhizosphere, and endosphere, for each method. Samples are displayed as dots (*n* = 10). **b** For each method, richness with increasing sequencing depth (*n* = 10) for each compartment of the plant dataset

Both Shannon diversity and richness were significantly lower in the ASV method than in the OTU and usASV methods, in accordance with the soil dataset but in contrast to the simulated and mock datasets (*P* < 0.001) (Fig. 6a, b). In most samples, the richness of the ASV and the usASV methods seemed to be correlated with the sequencing depth, but was less than for the bacterial soil dataset (Fig. 6b). The Shannon diversity and community richness was significantly higher in the rhizosphere compared to the endosphere for all methods (*P* < 0.05) (Fig. 6a, b).

The bacterial community structure between the rhizosphere and endosphere were further analyzed at family level to study the best-performing taxonomy based on our previous results. For the bacterial community structure analysis, the abundance filtered data (at least two counts per sample in at least four independent samples) were examined. Using the ASV, OTU, and usASV methods, 86, 168, and 157 families were detected, respectively (Fig. S3 and Table S3). Only one bacterial family, the *Rhodobiaceae,* was absent in the OTU method. The RA of this family, although still low, was remarkably higher in the usASV samples (0.11% ± 0.004 for the rhizosphere and 0.071% ± 0.02 for the endosphere) than in the ASV samples (0.041% ± 0.03 and 0.038% ± 0.02 for rhizosphere and endosphere, respectively) (Table S4). All remaining ASV families were detected in the other two methods as well. Similar to the soil dataset, the 22 unique OTU families absent in the ASV and the usASV methods were low abundant, with an RA below 0.5%. Additionally, 12 families were only detected by the usASV method and not by either the OTU or ASV methods. In total, 145 families were detected in the usASV and OTU methods, whereas only 85 of these occurred in the ASV method. These findings can be attributed to the same merging and filtering steps of USEARCH that were applied, instead of the ASV vs. OTU calling.

After identifying the families in the bacterial communities, differential abundances between rhizo- and endosphere samples were compared. The ASV method resulted in 32 families that were significantly enriched or depleted in the endosphere, in contrast to 151 and 83 differential families for the OTU and usASV methods, respectively (FDR < 0.05). In total, 24 families were significantly different in all three methods (FDR < 0.05). To illustrate the differences between the methods, we focused on the families *Erythrobacteraceae,* MND8*, Sphingomonadaceae*, and *Xanthomonadaceae*, which appeared to be depleted in the endosphere in all methods (Table S4). The detected depletion was significant (FDR < 0.05) in the ASV method for all four families, significant for only MND8 in the usASV method, with no significant depletion found at family level when analyzed using the OTU method (Table S4). The *Erythrobacteraceae* and MND8 families had both a relative abundance below 1% in all three methods in the rhizo- and endosphere, while the *Xanthomonadaceae* and the *Sphingomonadaceae* had an RA of > 1% (Table S4).

Three families that were significantly differential in the usASV method were completely absent in the OTU method. Furthermore, 17 other families were detected as significantly differential in the usASV method, but not in the OTU method. Except for three families, namely 0319_6G20, *Archangiaceae*, and MND8, all the significant families detected with the ASV method were also significant in the usASV method.

Most of the differences between the three methods were related to families with a very low RA. As for the soil dataset, low-abundant families were filtered out (RA < 0.1%; Table S3). The ASV method resulted in fewer than 50% of the families in comparison to the OTU table (86 vs. 186 families), while after the 0.5% filtering, 70% of the families counted in the OTU method were found in the ASV method (29 vs. 41 families). The trend of fewer ASVs than OTUs was also detected when the number of differentially abundant families was analyzed. Based on these results, we can conclude that the method used affected the discovered differences in RA between the rhizosphere and endosphere communities. The main differences of the methods were attributed to families with a low RA (< 0.1%).

### Differences between ASV and OTU methods in simulated fungal data, culture-based mock dataset and soil-related communities

To study the effect of methods on fungal datasets, we analyzed a simulated community, a culture-based mock and a soil-related fungal dataset using the three methods. Fungal datasets were simulated from the UNITE database (v7), accounting for differences in community richness and sequencing depth [25]. In addition, a fungal culture-based mock community containing 13 known plant pathogens was analyzed. Using the ASV method, the simulated fungal data had a higher diversity and richness than in the OTU method, which was again highly correlated with the sequencing depth (Fig. S4). However, in the fungal culture-based mock community, richness was overstated in the OTU method (102 ± 21) compared to richness in the ASV (15 ± 2) and usASV (35 ± 15), while at the same time the diversity of the OTU method was lower than the other two methods (Fig. S1C, D). Similar to the bacterial dataset, coverage for the simulated fungal dataset and culture-based mock was similar among all three methods, although with a slightly enhanced performance for the ASV method at the level of order and family in the simulated dataset (Fig. S4 and Table S1). The number of false positives was lower in the ASV method (five genera) than those in the OTU method (28 genera) and usASV (23 genera), which probably resulted in an overestimation of richness (Table S1).

In the soil-related dataset, the unfiltered ASV method provided half as many ASVs/OTUs as the OTU method did (2253 vs. 4438) (Table S2). After technical filtering, a decrease of approximately 70% for fungal ASVs was observed due to an elevated presence of unique sample sequences, which was similar to the bacterial ASVs. Results for the fungal alpha diversity were comparable with those in the bacterial communities for the three methods, with a significantly lower Shannon diversity and richness for the ASV method than for the OTU and usASV methods (*P* < 0.05) (Fig. S5 A, B). A plateau in fungal community richness and a richness-sequencing depth correlation were observed for the ASV method. A similar plateau was observed in the OTU method, with only a slight increase observed as sequencing depth increased. This disparity with the bacterial dataset is probably related to the lower richness of fungi in soil than bacteria. The usASV results were comparable to those of the ASV method.

When focusing on the structural composition, we found 75 fungal families that were all identified using the three methods after applying a more stringent filtering. In the OTU method, 50 additional fungal families were identified, most of which were absent in the ASV method due to the abundance filtering. Fourteen families were missing in the ASV dataset, but they were low-abundant in the OTU dataset (RA < 0.1%).

Analysis of the effect of tillage on the fungal family level (Fig. S5 C) revealed that in the ASV method, conventional tillage significantly changed the abundance of three families, with two families showing increased RA (FDR < 0.05). The same three families were also significantly affected in the OTU and usASV methods. Three additional significant families were found in the OTU method and seven extra families were found in the usASV method (FDR < 0.05).

In conclusion, the ASV method outperformed the OTU method when estimating the correct number of fungal species when the sample richness was low or when sufficient sequencing depth was present, possibly due to an increased number of false positives in the OTU method. In general, however, coverage was comparable. Differences between the two methods were found for low-abundant fungal families in the biological dataset, although this was less pronounced in comparison to the bacterial community.

## Discussion

Metabarcoding has become an essential tool to explore microbial communities in different environments. Increased analysis possibilities have resulted in the challenge of selecting the best method and correctly interpreting the results. We have shown that the choice between a clustering-based method (OTUs; USEARCH-UPARSE) and an error model-based method (ASVs; DADA2) affects the detection of microbial diversity, richness, composition, and differential abundances between treatments or sample types [12, 14], which inevitably leads to different biological conclusions. In this study we did not strive to compare all existing workflows, but rather to explore differences that occur when using either OTUs or ASVs on alpha and beta diversity parameters, and possible differences in biological conclusions made based on different outcomes in OTU and ASV analysis. For a methodological comparison of different OTU and ASV workflows we suggest reading [18, 20, 26].

To benchmark previous research, we first analyzed culture-based mock and simulated datasets. Here we studied the differences occurring due to the algorithm, rather than analyzing how the different methods cope with sequencing and PCR errors, therefore no errors were induced in the simulated dataset. Best results for both OTU and ASV methods were obtained for samples with high sequencing depth. Additionally, richness was best estimated by the ASV method. Based on the bacterial culture-based mock and simulated datasets, we can state that at family level, taxonomy was well determined (> 75% was correctly assigned), with low number of false positive and negative occurrences, in contrast to genus level (50% correct assignment). The underlying reasons of missing taxonomy is most likely different for each method. For the ASV method, the reason might be the high amount of unassigned (39.7% ± 6.1%) ASVs, whereas for the OTU method, it might be clustering into the most abundant sequence, which is not likely to be a biological sequence. Additionally, even though the databases are extensive, they most probably do not contain all sequences present in the culture-based mock.

The low number of correct taxonomy assignments could also be due to the technology applied, where only a short fragment of the 16S rRNA gene is amplified. To increase the taxonomic resolution, other marker genes such as GyraseB could be sequenced in parallel [27]. While the existing databases for marker genes other than the 16S rRNA gene are rather small, the distinctive capacity is better for several bacterial families. Other methods such as shotgun metagenomics studies or nanopore sequencing analysis can also be used to obtain results at lower taxonomic levels [28, 29].

For the comparison of metagenomics workflows, previous research focused strongly on strain detection within mock datasets, differences in alpha and beta diversities, and computing time [19, 20, 26, 30]. For research purposes, microbiomes are often analyzed at the biological level, because researchers are interested in examining either differential depletions and enrichments upon treatment or particular families with a certain RA. Therefore, we looked closely at whether the chosen methods also affects the bacterial and fungal community compositions, the differential abundances between treatments, and sample types for soil- and plant-related datasets, as these all may result in different biological conclusions.

In contrast to the community composition of the culture-based mock that was comparable when analyzed with ASV, OTU, or usASV methods, we detected unique families for each method in the soil- and plant-related datasets. Strikingly, the number of detected families (mostly low-abundant) with the OTU and usASV method was higher than that of the ASV method, hinting at a strong impact of the merging and filtering, which were similar for the OTU and usASV methods. Thus, when interested in low-abundant species, we recommend optimization of the merging and filtering settings of the chosen method.

To understand the differences in richness and diversity between OTU and ASV methods, we studied a simulated dataset with either low (100 or 500 independent species) or high (1000 or 2500 independent species) richness. For both ASV- and OTU calling, the estimation of richness and diversity was sufficient for low-diversity samples. This might be due to the inherent history of both methods, both of which have been optimized based primarily on low diverse datasets such as human-related microbiomes [11, 14]. The human gut is expected to be the most diverse human-related microbiome with an estimated richness of about 150–200 species [31]. In case of higher diversity in the sample, community richness and diversity are underestimated by the ASV and OTU methods, but the ASV method outperforms the OTU method when sequencing depth is high enough. Notably, a richness plateau appears for the simulated dataset in both the ASV and OTU methods. As no PCR or sequencing errors are introduced into this simulated dataset, the flattening of the curve can be due to better error capturing by the ASV method than the OTU clustering and therefore might also explain the lower diversity for the ASV method. Similar to the culture-based and simulated datasets, in both soil- and plant-related bacterial communities the diversity and number of detected ASVs were considerably lower than those of OTUs [20, 32]. The ASV richness seems to correlate with the sequencing depth, as the richness curve from the ASV method reaches a plateau, whereas this correlation was not detected in the OTU method. These observations can partially be explained by the read partitioning of the ASV error model, because the trends are the same for the usASV method, in which filtering and merging are done with USEARCH in combination with the error-calling model of DADA2. The detected reduced ASV diversity in the environmental datasets can thus partially be explained by a sequencing depth shortage that can be managed by sequencing fewer samples per run, although at an increased sequencing cost.

We have thus confirmed that richness is directly linked with the chosen method and strongly relates to sequencing depth. Estimation of the absolute number of species is still difficult based on metabarcoding data alone. Other approaches could be more informative, such as in-depth isolation techniques, cell counting with flow cytometry or the use of spike-in plasmids [33–35]. For metabarcoding data, we recommend studying relative changes in richness between treatments and samples rather than focus on absolute richness values. In our datasets, differences in diversities between rhizosphere and endosphere in the plant dataset could, for example, be detected in all methods. In addition, diversity measurements, such as the Shannon index, seem to be more consistent across the different methods.

Major differences were found between used methods in the significantly differential families upon treatment in the soil dataset and compartment in the plant dataset, most distinctly for low-abundant bacterial families. Even after filtering out families lower than 0.1 and 0.5% RA, the number of differential abundant families remained lower, although less pronounced, for the ASV than for the OTU method. Therefore, for the OTU-based analysis, stringent filtering during data analysis is advised for generating accurate results; the method is less effective in discriminating between biological variation and technical errors, and low-abundant families give uncertain results. This might cause the potential removal of valuable rare species, thereby reducing the sensitivity. Therefore, filtering should be considered independently for each research project. Some high abundant families also behaved differently between methods. For example, in the plant dataset, the *Xanthomonadaceae* and *Sphingomonadaceae* are significantly depleted in the endosphere when analyzed with the ASV or the usASV methods, but these high abundant families (> 4% in the rhizosphere and > 1.5% in the endosphere) are not detected as significant in the OTU methods.

Although bacterial richness and diversity are the main focus of microbiome analyses, fungi are also important ecological and functional players in soil- and plant related environments [36]. Fungal richness and diversity was studied here using the same pattern as for the three bacterial datasets (i.e., culture-based, mock and biological) used in this study. The number of ASVs show a correlation with diversity and, when the community is diverse enough, the richness is better estimated in the ASV method than in the OTU method. In contrast, the OTU method overestimated the richness in the fungal culture-based mock due to a higher number of false positives that were not observed in the bacterial culture-based mock. These observations can be linked to (i) insufficient management of the PCR and sequencing errors in the OTU method and (ii) the heterogeneous sequence lengths of the internal transcribed spacer (ITS) region for which the OTU clustering is not optimized. The differential analyses of the fungal soil dataset mainly revealed differences for the low-abundant families, although the treatment effect is less pronounced. Because the results of the OTU method and those of the usASV method are similar, the differences can be attributed to the filtering and merging of USEARCH. These findings suggest that the ASV method is better adapted to deal with fungal sequences as stated previously [19, 36].

In conclusion, caution is advised when analyzing metabarcoding data, because the results are affected by all of the steps of the workflow, starting from the sampling process through to the bioinformatic analysis. First, we have shown that the use of OTUs or ASVs will affect the biological conclusions drawn by the researcher. We have demonstrated this for the analysis and interpretation of low abundant taxa as different families will be classified depending on the choice of OTU or ASV method. This is also true for the alpha diversity measurements and the differential enriched or depleted families in environmental datasets. Based on our findings, we present four recommendations for metabarcoding analysis: (i) analyze and compare datasets at family level for best balance in data coverage and taxonomical relevance, (ii) use ASV levels when a high sequencing depth is guaranteed (more robust and trustworthy for bacterial sequences), (iii) study relative changes in richness values rather than absolute values, and (iv) use the ASV methods for fungal datasets (higher sensitivity, fewer false positives).

## Conclusions

The recent shift away from clustering metabarcoding datasets by OTUs toward ASVs affects the biological interpretation of data. We have made an in-depth comparison of both methods to distinguish the community composition and complexity. Both methods are generally established for low-complexity samples (e.g. human microbiome data) and bacterial communities. As most environmental samples are microbially very rich, we tested both bacterial and fungal communities in plant- and soil-related samples. Differences in differential abundances, richness and diversity were detected when applying the OTU vs. ASV method. These differences may result in different biological conclusions. The ASV method used outperformed the OTU method, but OTU is adequate to capture the community complexity. Additionally, we showed that analysis for each of the adopted methods is best performed at family level. Our results clearly indicate that caution is required when interpreting and analyzing metabarcoding data.

## Methods

### Datasets for simulated bacterial and fungal communities

Bacterial and fungal metabarcoding datasets were simulated using Grinder v0.5.4 [37]. For the bacterial and fungal datasets, the SILVA 16S rRNA gene (v132) database and UNITE (v7) were used as reference databases, respectively [22, 25]. In total, 100,000 sequences per dataset were randomly selected from the reference database based on a power-law abundance model to approximate true abundance models for metabarcoding libraries (a few high-abundant sequences and a high number of low abundance sequences), and the per-base quality was fixed on a Phred score of 30. From these sequences, the V3-V4 variable region of the 16S rRNA and ITS2 gene for the bacteria and fungi, respectively, were selected by an in silico PCR, with the primers of the field soil data set as input. In total, 20 datasets were created for bacteria and 20 for fungi. The dataset richness was equal to 100, 500, 1000, and 2500 in order to approximate low diversity communities (endosphere) and high diversity communities (soil). The five datasets per richness depth were rarefied to either 7500, 10,000, 20,000 or 50,000 or used as such without rarefaction to introduce differences into the sampling depth.

### Datasets for culture-based bacterial and fungal mock communities

To create the culture-based mock bacterial community with 254 bacterial isolates (Table S6 A), maize roots were sterilized and crushed [24]. Diluted suspensions were plated and incubated. Growing colonies were selected, streaked until pure cultures, and identified by Sanger sequencing based on the 16S rRNA gene with primers 27F and 1492R (Table S6 B) [38]. The bacterial strains were grown in liquid culture overnight with the same optical density (OD) and pooled (nonequimolar) (Table S6 A). Because closely related taxa are difficult to distinguish based on metabarcoding data, we deliberately included closely related strains into the bacterial mock community. DNA was extracted from the pooled bacterial culture and used six times as input for the metabarcoding library preparation.

The mock fungal community consisted of 13 fungal isolates, partly from ILVO’s fungal collection, and included members of the Ascomycota and Basidiomycota. DNA was pooled in equimolar concentrations in six-fold to obtain technical replicates (Table S6 C).

Six technical replicates per sample were used for metabarcoding of the bacterial and fungal mock communities. Illumina metabarcoding was done on the hypervariable V3-V4 region of the 16S rRNA gene and the ITS2 gene for the bacterial and fungal community, respectively, as described previously [39]. Libraries were sequenced using Illumina MiSeq v3 technology (2 × 300 bp, paired-end) by Admera Health (San Francisco, CA, USA) with 30% PhiX DNA as spike-in. The raw data were demultiplexed by the sequencing provider and primers were trimmed off with Trimmomatic (v0.32) [40].

### Biological datasets

Biological datasets were based on samples from a field trial of Flanders Research Institute for Agriculture, Fisheries and Food (ILVO) in Merelbeke, Belgium, according to applicable governmental rules and legislation [23]. In the field trial, soil was exposed to three treatments: (i) farm compost application (0 vs. 2000 kg C ha^− 1^ y^− 1^), (ii) tillage practices (conventional vs. non-inversion tillage), and (iii) slurry application (cattle vs. pig). The experimental set-up has been described previously [23]. Here, we focused only on the effect of the tillage practices, because a preliminary analysis showed that this treatment had the highest impact on the soil microbiome [41]. Topsoil (0–10 cm) was sampled in 2014 using an X sampling pattern resulting in eight technical sub-replicates that are mixed together to create one biological replicate. In total four biological replicates are present for each treatment. Samples were stored at − 20 °C and freeze-dried before DNA extraction from 250 mg soil using the DNeasy Powersoil Kit (Qiagen, Hilden, Germany) according to the manufacturer’s instructions. Illumina metabarcoding was done on the hypervariable V3-V4 (341 forward and 785 reverse primers) region of the 16S rRNA gene and the variable spacer of ITS2 gene (forward and reverse position depending on the fungal organism) for the bacterial and fungal communities, respectively, according to the described protocol [39] (Table S5). Libraries were sequenced by means of the Illumina MiSeq v3 technology (2 × 300 bp, paired-end) by the Oklahoma Medical Research Foundation (Oklahoma City, OK, USA) with 30% PhiX DNA as spike-in. The sequencing provider demultiplexed the raw data. Primers were trimmed off using Trimmomatic (v0.32) before further analysis [40].

For the plant-related experiment, seeds from one hybrid maize variety (LG30.217) were purchased (Limagrain, Saint Beauzire, France). All plants used in the experiments were grown in pots filled with Belgian field soil. The rhizosphere and endosphere of maize roots grown in field soil were sampled as described [24]. In brief, surface-sterilized maize seeds were sown in pots filled with Belgian field soil (sandy-loam soil; United State Department of Agriculture classification) (50°58′41″ N, 3°46′47.28″ E; Merelbeke, Belgium). Rhizosphere and endosphere were sampled after 3 weeks of growth under controlled growth chamber conditions (16 h/8 h light/dark regime, 21 °C). In total, 10 replicates were taken. Roots were shaken vigorously and washed briefly with phosphate-buffered saline (PBS) solution, the washing solutions were centrifuged (5 min at 10,000 g), and the remaining pellet was flash-frozen and designated as the rhizosphere samples. The washed roots were flash-frozen, designated as the endosphere samples, and ground before DNA extraction. DNA was isolated from all collected samples with the DNeasy PowerSoil DNA kit (Qiagen) and the V4 region (515 forward and 806 reverse primers) of the 16S rRNA gene was amplified (Table S5). An Illumina MiSeq platform (v3) was used according to the Illumina protocols for a 2 × 300-bp cycle run (VIB, Nucleomics Core, Leuven, Belgium). Demultiplexing was done by the sequencing provider and primers were removed using Trimmomatic (v0.32) [40].

### Downstream data analysis for the OTU workflow

Primer-free forward and reverse reads were merged with USEARCH (v2.6.0) [12]. For the field soil and mock communities, a quality threshold was set at 30 and a required minimal overlap size at 120 nucleotides for the V3-V4 (341 forward and 785 reverse primers) and ITS2 gene regions (forward and reverse position depending on the fungal organism) (Table S5). Minimal and maximal possible lengths of the assembled sequences was fixed at 400 and 450 nucleotides for the bacterial sequences and 200 to 450 nucleotides for the fungal sequences, respectively. For the pot experiment, the V4 region was amplified (515 forward and 806 reverse) (Table S5). Merging was set at a minimum overlap of 30 nucleotides. Subsequent analysis steps were the same for all experiments. All merged sequences were quality filtered with a maximum expected error of three with *fastx_filter* and sequences with uncalled bases were discarded with USEARCH (v2.6.0) [12]. The ITS2 region was extracted using ITSx (v1.1.1) [42]. The sequences of all samples were dereplicated and sorted by size. Reads were clustered into OTUs with UPARSE, with an identity level of 97 and 98.5% for bacterial and fungal sequences, respectively [21, 43]. Chimeras were removed with the *uchime_ref* command and the Gold ribosomal database project as a reference [44]. Sequences were mapped back to the representative OTUs with the *usearch_global* algorithm at 97% (bacterial) or 98.5% (fungal) identity and converted into an OTU table [45]. Taxonomy was assigned by means of *assignTaxonomy* of the DADA2 package using a naive Bayesian classifier method.

### Downstream data analysis for the ASV workflow

The DADA2 inference algorithm was used on primer-free reads to correct sequencing errors and create ASVs for the bacterial and fungal communities (v1.12) in R (v3.5.2) [14]. The reads were quality filtered by means of the *filterAndTrim* function that truncated the forward and reverse reads at 263 bp and 225 bp for the bacterial soil dataset and at 240 bp and 200 bp for the plant dataset, respectively, whereas a minimum length of 50 bp was used for the fungal soil dataset. For both bacterial and fungal datasets, reads were removed with more than three errors in the forward and five errors in the reverse reads. Reads were merged after inference of sequence variation with *learnerrors* and *dada* functions. Chimeric sequences were eliminated and taxonomy assigned with *assignTaxonomy* based on the SILVA database (v132) and UNITE (v7) databases for bacterial and fungal taxonomies, respectively [22, 46].

### Downstream data analysis for the usASV workflow

To determine the effect of filtering and merging on differences between workflows, we created the usASV workflow and tested it on the field soil dataset. Reads were merged and filtered as described for the OTU workflow. These filtered reads were then used to call ASVs and assign taxonomy by the DADA2 inference algorithm.

### Statistical analysis of the simulated and culture-based mock communities

For both the bacterial and fungal mock community datasets, two count tables were generated from the ASV and OTU workflows. For both count tables, only the ASVs/OTUs with a count of at least two in 1 M in at least three samples were kept for analysis. For each sample and each taxonomical level (kingdom, phylum, class, order, family, and genus), the number of true positives (TPs; taxa present in the mock community and correctly identified), false positives (FPs; taxa not originally present in the mock community, but identified in the analysis), and false negatives (FNs; taxa originally present in the mock community, but not identified in the analysis) were calculated. Based on these scores, we estimated the coverage for each taxonomical level by the following formula:

$$ \mathrm{Coverage}=\frac{True\ Positives}{Total\ expected\ result\ numbers} $$

In addition, the microbial richness, defined as the number of ASVs/OTUs recovered for each sample, was calculated at minimal sequencing depth. For all samples, convergence was reached based on the rarefaction plots (Fig. S1).

Metabarcoding communities and thus also mock communities suffer from biases introduced by sample handling and preservation, technical DNA extraction issues [47], and sequencing technology artefacts [48]. Therefore, to evaluate the ASV and OTU coverage for which no biases occurred, we generated in silico bacterial mock communities with a method similar to that described [49]. In brief, a binary classification test of TRUE or FALSE assignments per read was done that compared the taxonomy identification (taxid) of the expected lineage against the taxid of the taxonomic annotation at every taxonomic level from kingdom to genus. TP annotations were the TRUE ratings due to a correct taxonomic identification. When there was a misclassification, a FP was created, whereas a taxa that was incorrectly not classified (NA) was a FN. Coverage plots were obtained by taking into account each taxonomic level by means of the above formula. These coverage plots were made per richness values and for the two methods separately.

Besides these coverage plots, the richness was also calculated similarly as for the mock communities. These values were visualized by rarefaction plots. All analyses were done in R (v3.5.2) by means of the Phyloseq package (v3.10) and ggplot2 (v3.2.1) for graphical visualization [50].

### Statistical analysis of the biological datasets

For the soil and plant-related dataset, three count tables were generated: the ASV, OTU, and usASV workflows. A technical filtering of two counts in at least three independent samples was done on the bacterial and fungal datasets to remove spurious ASVs or OTUs and also mitochondrial and chloroplast sequences were removed in the bacterial datasets. Shannon-Wiener diversity indices were calculated with the microbiome package (v1.9.1) [51]. Because diversity and richness were not distributed normally, the means of the alpha diversity measurements were analyzed using the Kruskal-Wallis test followed with a pairwise Wilcoxon test and *p*-values were adjusted by the Holm-Bonferroni method. As no significant effect of treatments was detected for richness or Shannon diversity for the field soil, only pairwise comparison of the methods was done (*P* > 0.05). The biological analyses were done using Phyloseq (v3.10) and edgeR (v3.26.1) after abundance filtering of at least two counts per sample in at least four samples [50, 52]. Differential abundance in the soil microbiome was assessed for the effect of non-inversion tillage vs. conventional tillage on the family level by constructing the following model.

$$ E\ \left(\mathrm{RA}\right)\sim -1+\mathrm{Treatment}+{\mathrm{Block}}_{\mathrm{Field}} $$

The hypotheses of interest were tested by building a specific contrast, comparing the estimated mean differences in between tillage treatments. The data were normalized with the trimmed mean of M values (TMM) that corrects the effective library size of the count tables. For the soil dataset, a generalized linear model with negative binomial distribution (nbGLM) was applied to the counts for each ASV/OTU with tillage practices as main effect and a block effect to account for subplots on the field. For the plant dataset, the same model was used with the compartments as main effect and blocking on plants. The significance of the ASV or OTU changes was inferred with a quasi-likelihood F-test with a Benjamin-Hochberg False Discovery Rate [FDR] of 5% to adjust for multiple corrections. The plant dataset was analyzed similarly as the soil dataset. For the differential abundance analysis, the following model was used:

$$ E\ \left(\mathrm{RA}\right)\sim \mathrm{Compartment}+{\mathrm{Block}}_{\mathrm{Plant}} $$

The contrast of interest compared the mean differences of the rhizosphere and endosphere.

## Supplementary information

**Additional file 1: Figure S1.** Shannon diversity and richness versus sequencing depth of ASV, OTU and usASV method of a bacterial (A and B) and fungal (C and D) culture-based mock community. **A.** Shannon diversity of the bacterial culture-based mock community for each method. Technical replicates are displayed as dots (*n* = 6). **B.** For each method, richness of the bacterial culture-based mock community with increasing sequencing depth (until 80,000 sequences). **C.** Shannon diversity of the fungal culture-based mock community for each method. Technical replicates are displayed as dots (*n* = 6). **D.** For each method, richness of the fungal culture-based mock community with increasing sequencing depth (until 100,000 sequences). **Figure S2.** Differential abundances for non-inverted tillage versus conventional tillage in the soil dataset for bacterial families in all methods. All families found to be significant using the ASV, OTU, and usASV methods are shown with their respective relative abundance per treatment (*n* = 16). The presence of dots on the right indicates families with a significant effect of the applied treatment (FDR 5%) and the dot size corresponds to the logFC. The light and dark blue colors indicate decrease and increase in relative abundance, respectively. The gray boxes are families found to be significant in all three methods. **Figure S3.** Differential abundant bacterial families between rhizosphere and endosphere. All significant families found using the ASV, OTU, and usASV methods are shown with their respective relative abundance per compartment (*n* = 10). The presence of dots on the right of the box plots of the families indicate a significant effect of the applied treatment (FDR 5%) and the dot size represents the logFC. The light and dark blue colors indicates decrease and increase in the relative abundance, respectively. The gray boxes are families found to be significant in all three methods. **Figure S4.** Representation of the species richness, diversity, and coverage for the simulated fungal dataset analyzed either using the ASV method (red) or OTU method (blue). Datasets are simulated from the UNITE reference database (version 7) with an original community richness from 100 (light colored) and 2500 (dark colored). Top panel, Shannon diversity index per original sample richness; middle panel; community richness with increasing sequencing depth; and bottom panel, coverage of each method per taxonomic level. **Figure S5.** Shannon diversity and richness versus sequencing depth of ASV, OTU and usASV method in the fungal soil dataset (A and B) and differential abundances for non-inverted tillage versus plowing in the soil dataset for fungal families in all methods (C). **A.** Shannon diversity per treatment (treatments 1 and 2) for each method. Samples are displayed as dots (*n* = 16). **B.** Richness of each method with increasing sequencing depth. Sixteen replicates of each treatment are presented. **C**. All significant families found using the ASV, OTU, and usASV methods with their respective relative abundance per treatment (*n* = 16). The dots on the right of the boxplots indicate significant effects of the applied treatment (FDR 5%) and the dot size corresponds to the logFC. The light and dark blue colors indicate decrease and increase in the relative abundance, respectively. The gray boxes are families found to be significant in all three methods. **Table S1.** Differences in terms of coverage, number of false positives (FPs) and false negatives (FNs) between the ASV, OTU, and usASV methods in a bacterial and a fungal culture-based mock community. The bacterial mock community was assembled from plant-related species. In total, 254 bacterial species were present in the mock community, which could be classified to 4 phyla, 8 classes, 14 orders, 22 families, and 31 different genera based on the SILVA taxonomy (v132). The fungal mock community was assembled from plant-pathogenic species. In total, 14 fungal species were present in the mock community, which could be classified to 2 phyla, 5 classes, 9 orders, 12 families, and 12 genera based on the UNITE reference database. Six technical replicates were analyzed, each resulting in the same values for coverage, FPs, and FNs. **Table S2.** Number of ASVs/OTUs after each filtering step for all three methods per dataset. Applied filtering steps removed the chloroplast and mitochondrion sequences. Prevalence filtering was applied before the alpha diversity and abundance filtering before the differential abundance (DA) analysis. **Table S3.** Number of families and differentially abundant families after various filtering strategies for the ASV, OTU, and usASV methods for the soil and plant-related datasets. The families are counted after filtering out the low-abundant families (at least two CPM), families with a relative abundance higher than 0.1%, and those with one higher than 0.5%. The number of significantly differential families are also counted for the three filtering strategies (FDR < 0.05). **Table S4.** Relative abundances of five families in the OTU, ASV, and usASV methods. The relative abundances (% ± SE) of five families are presented. Asterisks indicate significant differences between rhizo- and endosphere (*, FDR < 0.5; **, FDR < 0.01). **Table S5**. Information of primer sets. **Table S6.** Dataset containing the bacterial and fungal collection used for the mock communities. **A.** List of all the bacterial strains with their assigned taxonomy using SILVA. **B.** 16S rRNA sequences of the bacterial strains of the mock community. **C**. Composition of the fungal mock community using the different DNA extraction methods.

## Data Availability

The raw demultiplexed sequence data are available in the NCBI Sequence Read Archive under accession numbers PRJNA602824 (field soil dataset), PRJNA524079 (plant-related experiment) and PRJNA601852 (bacterial and fungal mock) and PRJNA601863 (simulated bacterial and fungal mock). Scripts used to run all data analysis can be found at https://gitlab.com/lljoos/asv_vs_otu.

## Abbreviations

ASV: Amplicon Sequence Variant
DNA: Deoxyribonucleic Acid
FDR: False Discovery Rate
FN: False negative
FP: False positive
GLM: General linear model
nbGLM: negative binomial GLM
OTU: Operational Taxonomic Unit
RA: Relative abundance
RNA: Ribonucleic Acid
SE: Standard error
TMM: Trimmed mean of M values
TP: True positive
