# Supplementary material for: Daring to be differential: metabarcoding analysis of soil and plant-related microbial communities using amplicon sequence variants and operational taxonomical units
Source: BMC Genomics. 2020 Oct 22;21:733. doi: 10.1186/s12864-020-07126-4 (PMC7579973; doi:10.1186/s12864-020-07126-4)
Supplement: Supplementary file 1 — Additional file 1: Figure S1. Shannon diversity and richness versus sequencing depth of ASV, OTU and usASV method of a bacterial (A and B) and fungal (C and D) culture-based mock community. A. Shannon diversity of the bacterial culture-based mock community for each method. Technical replicates are displayed as dots (n = 6). B. For each method, richness of the bacterial culture-based mock community with increasing sequencing depth (until 80,000 sequences). C. Shannon diversity of the fungal culture-based mock community for each method. Technical replicates are displayed as dots (n = 6). D. For each method, richness of the fungal culture-based mock community with increasing sequencing depth (until 100,000 sequences). Figure S2. Differential abundances for non-inverted tillage versus conventional tillage in the soil dataset for bacterial families in all methods. All families found to be significant using the ASV, OTU, and usASV methods are shown with their respective relative abundance per treatment (n = 16). The presence of dots on the right indicates families with a significant effect of the applied treatment (FDR 5%) and the dot size corresponds to the logFC. The light and dark blue colors indicate decrease and increase in relative abundance, respectively. The gray boxes are families found to be significant in all three methods. Figure S3. Differential abundant bacterial families between rhizosphere and endosphere. All significant families found using the ASV, OTU, and usASV methods are shown with their respective relative abundance per compartment (n = 10). The presence of dots on the right of the box plots of the families indicate a significant effect of the applied treatment (FDR 5%) and the dot size represents the logFC. The light and dark blue colors indicates decrease and increase in the relative abundance, respectively. The gray boxes are families found to be significant in all three methods. Figure S4. Representation of the species richness, diversity, and c [file 12864_2020_7126_MOESM1_ESM.zip › 12864_2020_7126_MOESM1_ESM.docx]

**SUPPLEMENTAL MATERIAL**


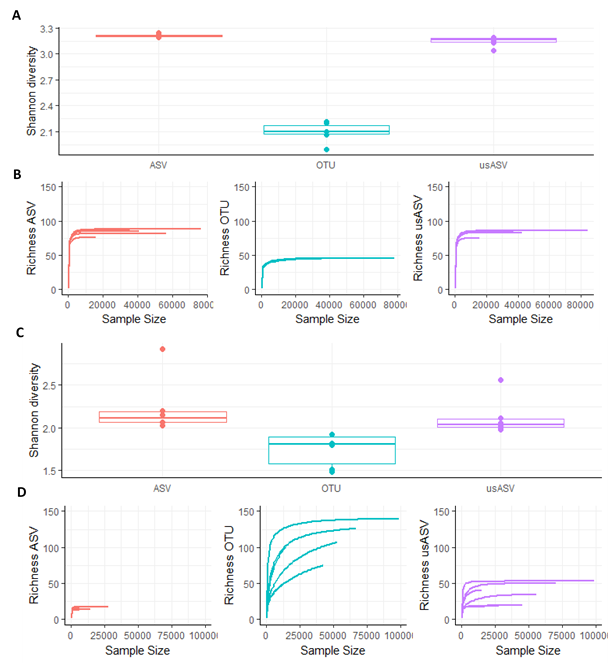


**Figure S1.** Shannon diversity and richness versus sequencing depth of ASV, OTU and usASV method of a bacterial (A and B) and fungal (C and D) culture-based mock community. **A.** Shannon diversity of the bacterial culture-based mock community for each method. Technical replicates are displayed as dots (n = 6). **B.** For each method, richness of the bacterial culture-based mock community with increasing sequencing depth (until 80,000 sequences). **C.** Shannon diversity of the fungal culture-based mock community for each method. Technical replicates are displayed as dots (n = 6). **D.** For each method, richness of the fungal culture-based mock community with increasing sequencing depth (until 100,000 sequences).


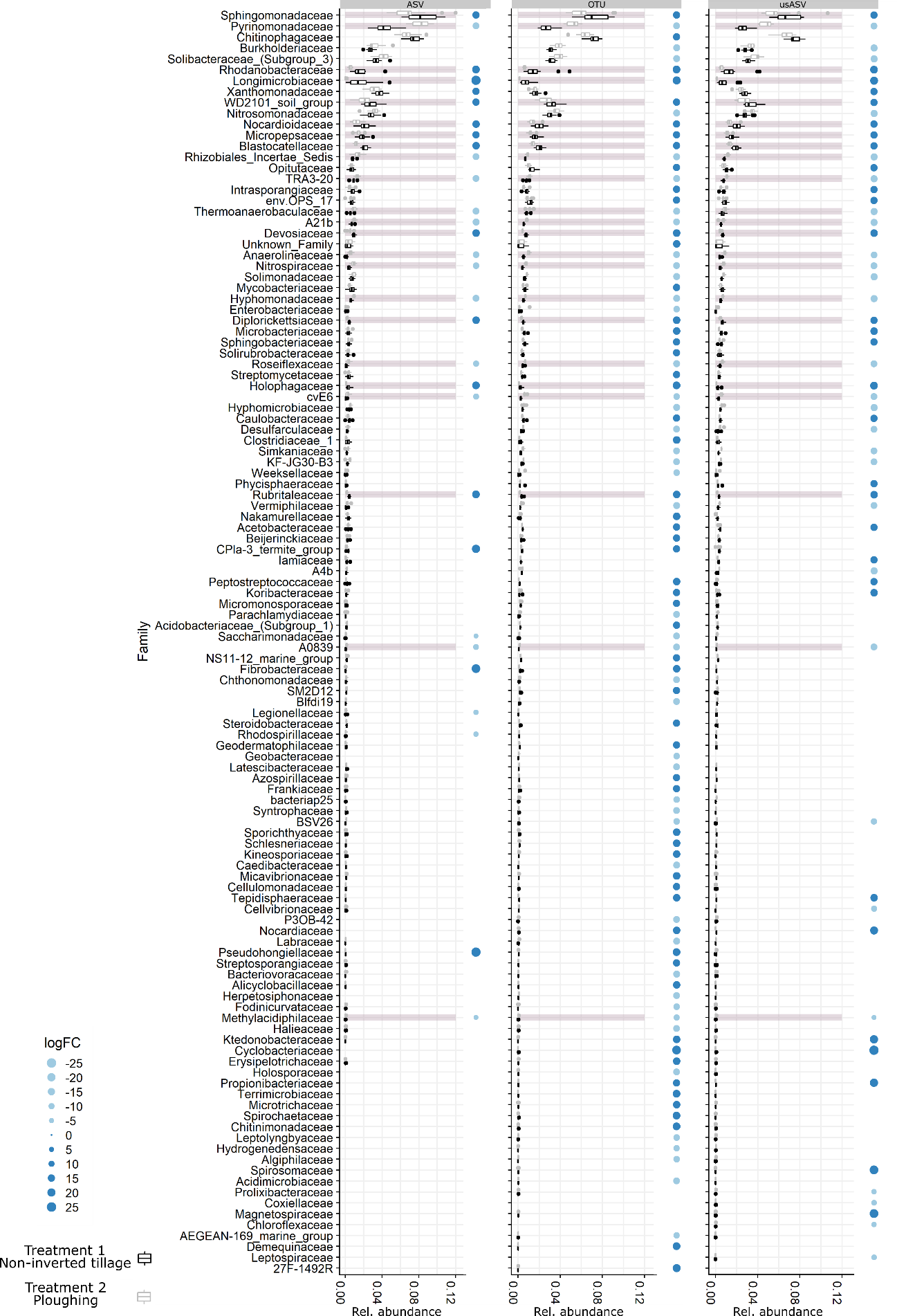


**Figure S2.** Differential abundances for non-inverted tillage versus conventional tillage in the soil dataset for bacterial families in all methods. All families found to be significant using the ASV, OTU, and usASV methods are shown with their respective relative abundance per treatment (n = 16). The presence of dots on the right indicates families with a significant effect of the applied treatment (FDR 5%) and the dot size corresponds to the logFC. The light and dark blue colors indicate decrease and increase in relative abundance, respectively. The gray boxes are families found to be significant in all three methods.

**
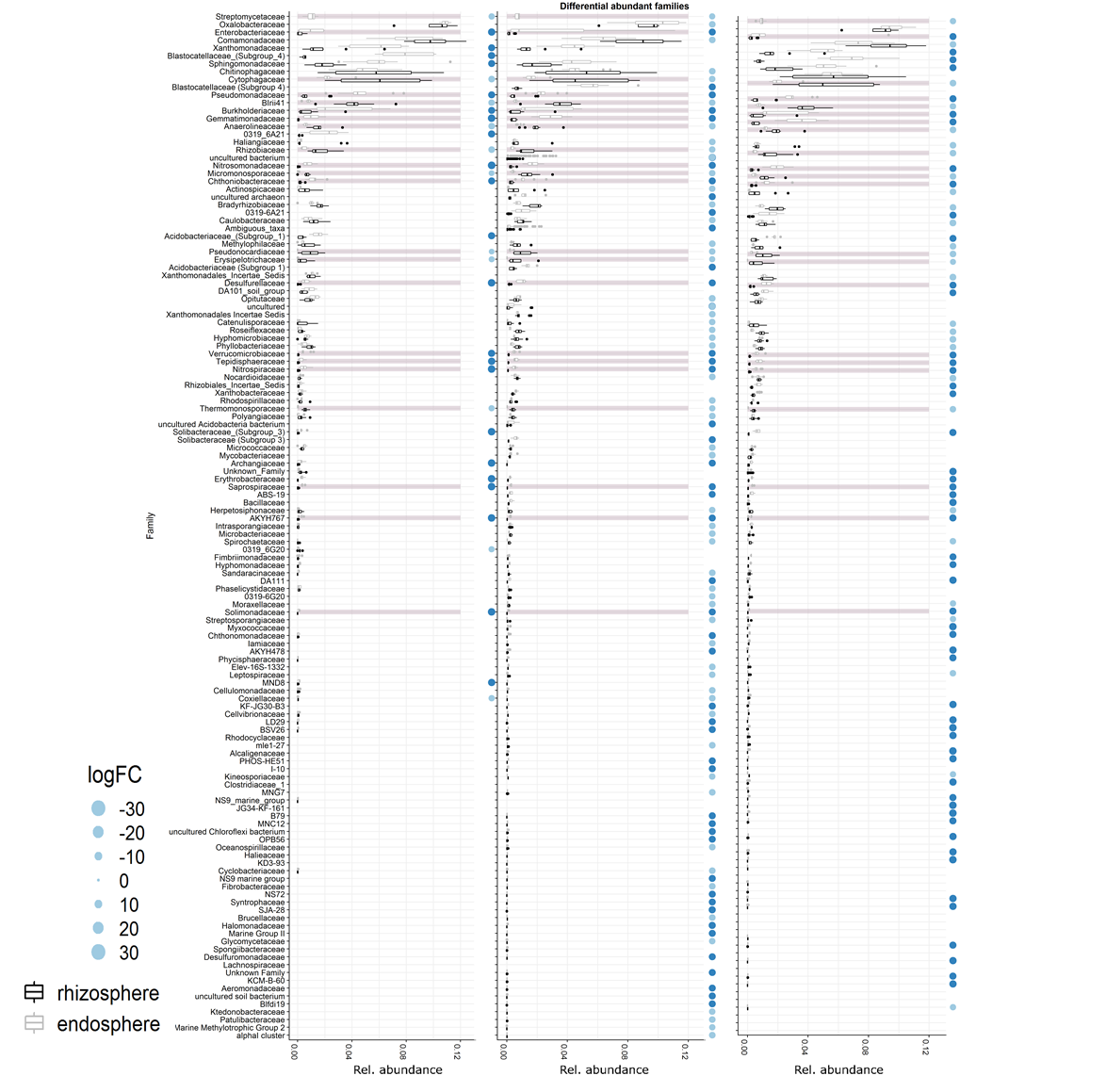
**

**Figure S3.** Differential abundant bacterial families between rhizosphere and endosphere. All significant families found using the ASV, OTU, and usASV methods are shown with their respective relative abundance per compartment (n = 10). The presence of dots on the right of the box plots of the families indicate a significant effect of the applied treatment (FDR 5%) and the dot size represents the logFC. The light and dark blue colors indicates decrease and increase in the relative abundance, respectively. The gray boxes are families found to be significant in all three methods.

**
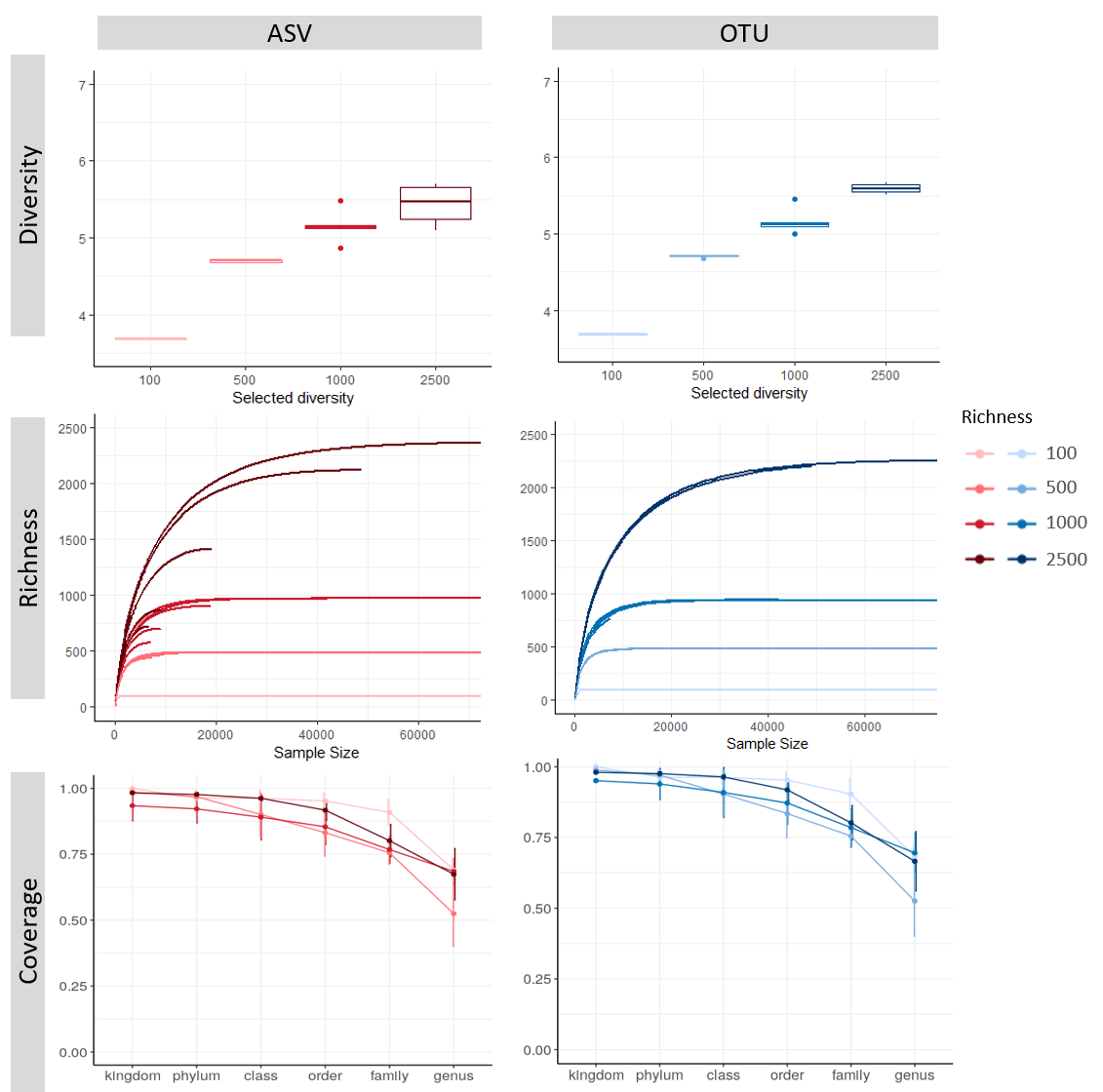
**

**Figure S4.** Representation of the species richness, diversity, and coverage for the simulated fungal dataset analyzed either using the ASV method (red) or OTU method (blue). Datasets are simulated from the UNITE reference database (version 7) with an original community richness from 100 (light colored) and 2,500 (dark colored). Top panel, Shannon diversity index per original sample richness; middle panel; community richness with increasing sequencing depth; and bottom panel, coverage of each method per taxonomic level.


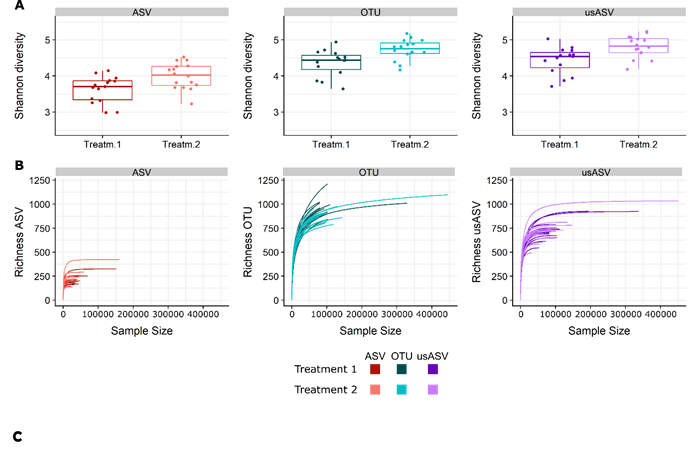


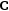

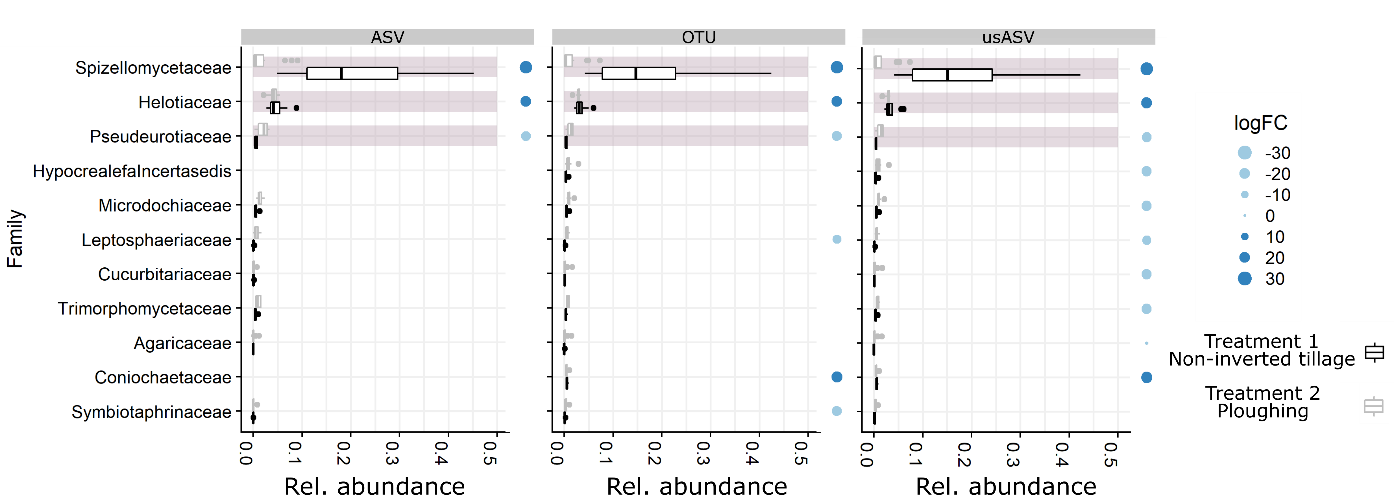


**Figure S5.** Shannon diversity and richness versus sequencing depth of ASV, OTU and usASV method in the fungal soil dataset (A and B) and differential abundances for non-inverted tillage versus plowing in the soil dataset for fungal families in all method s(C). **A.** Shannon diversity per treatment (treatments 1 and 2) for each method. Samples are displayed as dots (n = 16). **B.** Richness of each method with increasing sequencing depth. Sixteen replicates of each treatment are presented. **C**. All significant families found using the ASV, OTU, and usASV methods with their respective relative abundance per treatment (n = 16). The dots on the right of the boxplots indicate significant effects of the applied treatment (FDR 5%) and the dot size corresponds to the logFC. The light and dark blue colors indicate decrease and increase in the relative abundance, respectively. The gray boxes are families found to be significant in all three methods.

**Table S1.** Differences in terms of coverage, number of false positives (FPs) and false negatives (FNs) between the ASV, OTU, and usASV methods in a bacterial and a fungal culture-based mock community. The bacterial mock community was assembled from plant-related species. In total, 254 bacterial species were present in the mock community, which could be classified to 4 phyla, 8 classes, 14 orders, 22 families, and 31 different genera based on the SILVA taxonomy (v132). The fungal mock community was assembled from plant-pathogenic species. In total, 14 fungal species were present in the mock community, which could be classified to 2 phyla, 5 classes, 9 orders, 12 families, and 12 genera based on the UNITE reference database. Six technical replicates were analyzed, each resulting in the same values for coverage, FPs, and FNs.

| **Bacteria** | **Coverage** | | | **False positives** | | | **False negatives** | | |
| --- | --- | --- | --- | --- | --- | --- | --- | --- | --- |
|  | **ASV** | **OTU** | **usASV** | **ASV** | **OTU** | **usASV** | **ASV** | **OTU** | **usASV** |
| **Kingdom** | 1/1 | 1/1 | 1/1 | 0 | 0 | 0 | 0 | 0 | 0 |
| **Phylum** | 4/4 | 4/4 | 4/4 | 0 | 1 | 0 | 0 | 0 | 0 |
| **Class** | 7/8 | 7/8 | 7/8 | 1 | 2 | 1 | 1 | 1 | 1 |
| **Order** | 12/14 | 12/14 | 12/14 | 1 | 2 | 1 | 2 | 2 | 2 |
| **Family** | 15/22 | 15/22 | 15/22 | 3 | 6 | 3 | 7 | 7 | 7 |
| **Genus** | 21/31 | 22/31 | 22/31 | 11 | 8 | 13 | 10 | 9 | 9 |
| **Fungi** |  |  |  |  |  |  |  |  |  |
| **Kingdom** | 1/1 | 1/1 | 1/1 | 0 | 0 | 0 | 0 | 0 | 0 |
| **Phylum** | 2/2 | 2/2 | 2/2 | 0 | 3 | 3 | 0 | 0 | 0 |
| **Class** | 4/5 | 4/5 | 4/5 | 2 | 6 | 2 | 1 | 1 | 1 |
| **Order** | 7/9 | 8/9 | 8/9 | 2 | 15 | 12 | 2 | 1 | 1 |
| **Family** | 10/12 | 11/12 | 11/12 | 3 | 27 | 21 | 2 | 1 | 1 |
| **Genus** | 8/12 | 8/12 | 8/12 | 5 | 28 | 23 | 4 | 4 | 4 |

**Table S2.** Number of ASVs/OTUs after each filtering step for all three methods per dataset. Applied filtering steps removed the chloroplast and mitochondrion sequences. Prevalence filtering was applied before the alpha diversity and abundance filtering before the differential abundance (DA) analysis.

| **Field soil dataset – bacterial community** | | | |
| --- | --- | --- | --- |
|  | **ASV workflow** | **OTU workflow** | **usASV workflow** |
| Before filtering | 11,492 | 13,407 | 21,282 |
| Removed chloroplast and mitochondrion | 11,402 | 13,263 | 21,138 |
| Technical filtering (< 2 counts in three independent samples) | 3,792 | 11,613 | 7,114 |
| Abundance filtering (< 2 CPM in four independent samples) | 2,278 | 2,775 | 4,981 |
| **Plant dataset – bacterial community** | | | |
|  | **ASV workflow** | **OTU workflow** | **usASV workflow** |
| Before filtering | 4,839 | 11,626 | 13,347 |
| Removed chloroplast and mitochondrion | 4,602 | 11,355 | 13,012 |
| Technical filtering (< 2 counts in three independent samples) | 734 | 5,782 | 25,21 |
| Abundance filtering (< 2 CPM in four independent samples) | 552 | 1,822 | 1,860 |
| **Field soil dataset – fungal community** | | | |
|  | **ASV workflow** | **OTU workflow** | **usASV workflow** |
| Before filtering | 2,253 | 4,438 | 6,361 |
| Technical filtering (< 2 counts in three independent samples) | 544 | 2,382 | 1,749 |
| Abundance filtering (< 2 CPM in four independent samples) | 390 | 1,082 | 1,144 |

**Table S3.** Number of families and differentially abundant families after various filtering strategies for the ASV, OTU, and usASV methods for the soil and plant-related datasets. The families are counted after filtering out the low-abundant families (at least two CPM), families with a relative abundance higher than 0.1%, and those with one higher than 0.5%. The number of significantly differential families are also counted for the three filtering strategies (FDR < 0.05).

|  | **ASV** | **OTU** | **usASV** |
| --- | --- | --- | --- |
| **Field soil dataset – bacterial community** | | | |
| 2 cpm filtering | | | |
| Number of families | 148 | 188 | 196 |
| Number of significantly different families | 30 | 104 | 58 |
| > 0.1% filtering | | | |
| Number of families | 90 | 102 | 101 |
| Number of significantly different families | 30 | 61 | 51 |
| > 0.5% filtering | | | |
| Number of families | 45 | 51 | 50 |
| Number of significantly different families | 18 | 30 | 30 |
| **Plant dataset – bacterial community** | | | |
| 2 cpm filtering | | | |
| Number of families | 86 | 168 | 157 |
| Number of significantly different families | 32 | 157 | 83 |
| > 0.1% filtering | | | |
| Number of families | 60 | 75 | 73 |
| Number of significantly different families | 29 | 67 | 46 |
| > 0.5% filtering | | | |
| Number of families | 29 | 41 | 38 |
| Number of significantly different families | 16 | 34 | 27 |

**Table S4.** Relative abundances of five families in the OTU, ASV, and usASV methods. The relative abundances (% ± SE) of five families are presented. Asterisks indicate significant differences between rhizo- and endosphere (*, FDR < 0.5; **, FDR < 0.01). **Table S5**. Information of primer sets.

|  | **ASV** | | **OTU** | | **usASV** | |
| --- | --- | --- | --- | --- | --- | --- |
|  | **Rhizosphere** | **Endosphere** | **Rhizosphere** | **Endosphere** | **Rhizosphere** | **Endosphere** |
| *Rhodobiaceae* | 0.041 ± 0.028 | 0.038 ± 0.021 | // | // | 0.11 ± 0.004 | 0.071 ± 0.019 |
| *Erythrobacteraceae* | 0.32 ± 0.044 | 0.022 ± 0.011 (**) | 0.22 ± 0.022 | 0.074 ± 0.012 | 0.29 ± 0.012 | 0.067 ± 0.014 (*) |
| *Sphingomonodaceae* | 6.05 ± 0.81 | 1.94 ± 0.32 (*) | 4.48 ± 0.54 | 1.96 ± 0.35 | 5.20 ± 0.35 | 2.09 ± 0.32 (*) |
| *Xanthomonadaceae* | 6.97 ± 1.34 | 1.88 ± 0.58 (**) | 4.28 ± 0.51 | 1.64 ± 0.41 | 5.74 ± 0.43 | 1.84 ± 0.40 (**) |
| MND8 | 0.1 ± 0.027 | 0.004 ± 0.004 (*) | 0.04 ± 0.005 | 0.012 ± 0.002 | 0.068 ± 0.005 | 0.014 ± 0.005 |

**Table S5.** Information of primer sets.

| **Purpose** | **Primer name** | **Primer sequence (5’-3’)** | **Direction** | **Reference** |
| --- | --- | --- | --- | --- |
| Bacterial mock | 27F | AGAGTTTGATCMTGGCTCAG | Forward | Niemann et al. (1997) |
|  | 1492R | GGTTACCTTGTTACGACTT | Reverse |  |
| Fungal mock | ITS1  ITS4 | TCCGTAGGTGAACCTGCGG  TCCTCCGCTTATTGATATGC | Forward  Reverse | White et al. (1990) |
| Metabarcoding V3-V4 region 16S rRNA gene | S-D-Bact-0341-b-S-17 | CCTACGGGNGGCWGCAG | Forward | Klindworth et al. (2013) |
|  | S-D-Bact-0785-a-A-21 | GACTACHVGGGTATCTAATCC | Reverse |  |
| Metabarcoding ITS2 gene | fITS7bis - adapted | GTGAATCATCRAATYTTTG | Forward | Ihrmark et al. (2012) |
|  | ITS4NGSr | TCCTSCGCTTATTGATATGC | Reverse | Tedersoo et al. (2014) |
| Metabarcoding V4 region 16S rRNA gene | 515F | GTGYCAGCMGCCGCGGTAA | Forward | Caporaso et al. (2011) |
|  | 806R | GGACTACNVGGGTWTCTAAT | Reverse |  |

Caporaso, J.G., Lauber, C.L., Walters, W.A., Berg-Lyons, D., Lozupone, C.A., Turnbaugh, P.J., Fierer, N., and Knight, R. (2011). Global patterns of 16S rRNA diversity at a depth of millions of sequences per sample. Proc. Natl. Acad. Sci. USA 108, 4516-4522.

Ihrmark, K., Bödeker, I., Cruz-Martinez, K., Friberg, H., Kubartova, A., Schenck, J., Strid, Y., Stenlid, J., Brandström-Durling, M., Clemmensen, K.E., and Lindahl, B.D. (2012). New primers to amplify the fungal ITS2 region–evaluation by 454-sequencing of artificial and natural communities. FEMS Microbiol. Ecol. 82, 666-677.

Klindworth, A., Pruesse, E., Schweer, T., Peplies, J., Quast, C., Horn, M., and Glöckner, F.O. (2013). Evaluation of general 16S ribosomal RNA gene PCR primers for classical and next-generation sequencing-based diversity studies. Nucleic Acids Res. 41, e1.

Niemann, S., Pühler, A., Tichy, H.-V., Simon, R., and Selbitschka, W. (1997). Evaluation of the resolving power of three different DNA fingerprinting methods to discriminate among isolates of a natural *Rhizobium meliloti* population. J Appl Microbiol. 82, 477-484.

Tedersoo, L., Bahram, M., Põlme, S., Kõljalg, U., Yorou, N.S., Wijesundera, R., Ruiz, L.V., Vasco-Palacios, A.M., Thu, P.Q., Suija, A., Smith, M.E., Sharp, C., Saluveer, E., Saitta, A., Rosas, M., Riit, T., Ratkowsky, D., Pritsch, K., Põldmaa, K., Piepenbring, M., Phosri, C., Peterson, M., Parts, K., Pärtel, K., Otsing, E., Nouhra, E., Njouonkou, A.L., Nilsson, R.H., Morgado, L.N., Mayor, J., May, T.W., Majuakim, L., Lodge, D.J., Lee, S.S., Larsson, K.-H., Kohout, P., Hosaka, K., Hiiesalu, I., Henkel, T.W., Harend, H., Guo, L.-d., Greslebin, A., Grelet, G., Geml, J., Gates, G., Dunstan, W., Dunk, C., Drenkhan, R., Dearnaley, J., De Kesel, A., Dang, T., Chen, X., Buegger, F., Brearley, F.Q., Bonito, G., Anslan, S., Abell, S., and Abarenkov, K. (2014). Global diversity and geography of soil fungi. Science 346, 1256688.

White, T.J., Bruns, T., Lee, S., and Taylor, J. (1990). Amplification and direct sequencing of fungal ribosomal RNA genes for phylogenetics. In PCR Protocols: a Guide to Methods and Applications, M.A. Innis, D.H. Gelfand, J.J. Sninsky, and T.J. White, eds. (San Diego: Academic Press), pp. 315-322.

**Table S6.** Dataset containing the bacterial and fungal collection used for the mock communities. **A.** List of all the bacterial strains with their assigned taxonomy by means of SILVA. **B.** 16S rRNA sequences of the bacterial strains of the mock community. **C**. Composition of the fungal mock community with respective DNA extraction methods.
